# Natural Antisense Transcript: A Concomitant Engagement with Protein-Coding Transcript

**DOI:** 10.18632/oncotarget.178

**Published:** 2010-10-09

**Authors:** Keguo Li, Ramani Ramchandran

**Affiliations:** Division of Developmental Biology, Developmental Vascular Biology Program, Department of Pediatrics, Children's Research Institute, Medical College of Wisconsin, Milwaukee, WI, USA

**Keywords:** Non-coding RNA, antisense, zebrafish, tie-1

## Abstract

The vertebrate genome contains large spans of non-coding RNA, which for the most part were considered of little functional value to the organism. Recent studies have indicated that vertebrate genomes may have stored hidden secrets in this large span of non-coding RNA, which we refer to here as “Natural Antisense Transcripts (NATs).” NATs can be found in introns, exons, promoters, enhancers, intergenic sequences, and untranslated regions of the genome. They can be located in either the plus or minus DNA strand. NATs utilize several mechanisms that include DNA replication interference, chromatin remodeling, transcriptional interference, RNA masking, double-stranded RNA (dsRNA)-dependent mechanisms and translation interference to mechanistically regulate gene expression. Recently, NAT levels have been identified as dysregulated in various disease states. This review presents an overview of the current state of NAT biology and highlights the main points with specific examples.

## THE PREVALENCE OF NATURAL ANTISENSE TRANSCRIPT (NAT) IN VERTEBRATES

Natural antisense transcript (NAT) is defined as any RNA transcript that is complementary to an endogenous RNA transcript [1]. Our knowledge about NAT began from viral transcripts. In 1969, Bovre K et al found the central b2 region in the coliphage λ genome could produce two opposite oriented mRNA, one originating on the plus strand and the other on the minus strand, which partially overlap with each other [2]. Later, similar transcriptional events were identified in prokaryotes [3] and eukaryotes [4]. Systematic identification of NATs in mammalian cells began in 2002 utilizing a microbead capture double strand cDNA strategy [5]. Genome-wide analyses show that more than 63% of transcripts have antisense (AS) partners, most of which represent non-protein-coding RNAs [6]. In human genome, 22% of the human transcription clusters form sense (S)/antisense (AS) pairs. Through in-depth analysis of the functional elements in the human genome (1% coverage), the Encyclopedia of the DNA element (ENCODE) pilot project discovered that almost all DNA is transcribed into RNA, producing a large number of overlapping transcripts [7]. Several methods are utilized to identify NATs and are reviewed elsewhere [8]. Because NATs are found in low abundance, they are often lost in transcriptome analysis. Analysis of 29 human LongSAGE libraries demonstrated that antisense tag only contributes 8% of the total transcript copies [9]. The percentage of NATs is 2.8% in worm, 11% in yeast and 12% in malaria [10-12]. With the completed sequencing of the human genome, the gene numbers has been revised to 25,000 [13] because more transcripts continue to be discovered, many of which do not correspond to annotated genes, but to intergenic and intronic regions. Most AS transcripts do not have protein-coding capacity [14]. At present, the Unigene database of the National Center for Biotechnology Information indicates 123,200 entries for human and 79,222 for mouse. To date, protein-coding transcripts just account for ~1.5% genome, and the remainder transcribe into a large amount of non-coding RNAs, of which NATs are dominant [13].

## UNDERLYING MECHANISMS OF NAT FUNCTION

Most S/AS transcript pairs are conserved in evolution and are concurrently or inversely expressed in a cell suggesting a correlative functional role. Six mechanisms widely dictate how NAT's function: (a) DNA replication interference, (b) chromatin remodeling, (c) transcriptional interference, (d) RNA masking, (e) double-stranded RNA (dsRNA)-dependent mechanisms and (f) translation interference [15]4. We will discuss the general principles of each NAT mechanism followed by a specific example.

### (a) DNA replication interference

DNA replication can be interfered and thereby inhibited by NATs. This was demonstrated elegantly in the *E. coli* plasmid ColE1. The ColE1 primer transcript (RNA II) binds to the template DNA near replication origin. The plasmid replication is dependent on the primer formation (RNA II). Hybridization of RNA II to DNA is inhibited when a second RNA molecule RNA 1 (NAT RNA) binds to RNA II, which results in the alteration of secondary structure of RNA II [12].

### (b) Chromatin Remodeling

Chromatin remodeling is generally defined as any event that alters the nuclease sensitivity of a region of chromatin. These events can occur independently or in concert with other events, such as transcription [16]. S/AS pairs are especially abundant in imprinted loci, suggesting the putative role of non-coding RNA in gene silencing [6]. For example, in leukemia, an inverse relationship was observed between p15 antisense (p15AS) and p15 sense expression. The p15AS induces p15 silencing *in cis* and *in trans* through heterochromatin formation in leukemic cells and in mouse ES cells.

### (c) Transcriptional interference

Transcriptional interference refers to the direct negative impact of one transcriptional activity on a second transcriptional activity *in cis*. This happens through 3 kinds of mechanisms: (1) occlusion in which the passage of elongating RNA polymerases (RNAPs) blocks the access to the promoter; (2) collisions between elongating RNAPs, moving in opposite directions, leading to premature termination of transcription; and (3) ‘sitting duck' interference which refers to the removal of promoter-bound complexes by the passage of RNAPs from the opposing promoter [17]. A non-translated AS RNA (RnaG) transcribes *in cis* on the complementary strand of *icsA*, a virulence gene of *Shigella flexneri*. The *icsA* and *RnaG* promoters are in opposite direction and 120 bp apart. *In vitro* transcription from the strong *RnaG* promoter (aggressive) dramatically inhibits transcription from the weaker *icsA* promoter (sensitive) [18].

### (d) RNA masking

RNA duplex formation may mask critical regulatory features within transcripts, thereby inhibiting the binding of other trans-acting factors. RNA masking intervenes mRNA splicing, transport, polyadenylation, translation and degradation [19]. Zeb2 NAT involved in epithelial—mesenchymal transition (EMT) uses this mechanism. Efficient translation of Zeb2 does not depend on 5' cap structure, but on internal ribosome entry site (IRES) embedded in an intron sequence of Zeb2 mRNA. In epithelial cells, Zeb2 mRNA is fully spliced and cannot be translated. During EMT, Zeb2 NAT complements with the 5' splice site of the intron containing IRES in the 5' UTR of Zeb2, which prevents the splicing of the intron required for efficient translation and expression of the Zeb2 protein [20].

### (e) Double stranded RNA-dependent mechanism

Duplex RNA can be recognized by RNAi machinery. These dsRNAs are rapidly processed into short RNA duplexes of 21 to 28 nucleotides in length, which then guide the recognition and ultimately the cleavage or translational repression of complementary single-stranded RNAs, such as messenger RNAs or viral genomic/anti-genomic RNAs. In a recent experiment in *X. Laevis*, double stranded Slc34a/antisense Slc34a injected into the nucleus of *xenopus* oocytes were degraded into short RNAs of ~23 bases within 4 h [21]. Similarly, more than 100 NATs have been processed into siRNA in *D. melanogaster* [10].

### (f) Translation interference

NATs can also prevent transcripts from translating by simply interfering with the translation apparatus. Antisense PU.1 regulates PU.1 expression level via translation interference. The absolute levels of PU.1, a transcription factor required for normal hematopoiesis is critical for specifying cell fate, and, if perturbed, even modest decreases in PU.1 can lead to leukemias and lymphomas. Antisense PU.1 forms complex with three translation factors eIF4A, eEF1A and 4E-BP1. The complex stalls translation between the initiation and elongation steps [22].

## NATS ROLE IN PATHOLOGY

Although the physiological level of NATs is very low, their levels go up dramatically in disease. For example, β-secretase-1 (*BACE1*) is a crucial enzyme in Alzheimer's disease pathophysiology and antisense *BACE1 (BACE1*-AS) is markedly up-regulated in brain samples from Alzheimer's disease patients [23]. Similarly, antisense ApoE is 100-fold up-regulated in spinal cord-injured C57BL/6 mice compared with normal levels [24].

Tumor pathology often observes AS RNA level changes and in turn shows aberrant regulation of cognate transcripts. In non-papillary clear-cell renal tumors, the NAT of hypoxia inducible factor α (HIFα) increases 10- to 100-fold [25]. Interestingly, a positive correlation of differential expression was observed for most sense and antisense transcript pairs between normal and malignant breast samples [26]. This suggests that aberrant expression of NATs may provide value as a diagnostic reagent. For neuroblastoma (NB), pediatric brain tumor condition, the ratio of antisense MYCN to MYCN is directly correlated with NB disease stage. In the more advanced NB stages and NBs with MYCN-amplification, relatively more MYCN-AS is present as compared to MYCN. Further, expression of the antisense gene MYCN-AS is speculated to be relevant to the progression of NB [27].

Similar to tumor studies, the relevance of AS RNA levels to progression of cardiovascular disease is unknown. In a recent study, the analysis in peripheral blood mononuclear cells from 1098 patients of coronary artery disease demonstrated positive correlation of AS noncoding RNA in the INK4 locus (ANRIL) expression with atherosclerosis [28]. Our recent study on non-coding RNA identified a natural AS transcript for tyrosine kinase containing immunoglobulin and epidermal growth factor homology domain-1 (*tie-1*), *tie-1AS* lncRNA in zebrafish, mouse and humans. In embryonic zebrafish, *tie-1AS* lncRNA transcript is expressed temporally and spatially *in vivo* with its native target, the *tie-1* coding transcript and in additional locations (ear & brain). This argues for a *tie-1* gene role previously unrecognized in the brain and ear function, perhaps in the vasculature of these organs. In fact two recent studies implicate Angiopoietins, ligands for Tie's, in lymphatic vessel development in ear [29,30]. Since the non-coding RNA for *tie-1* is conserved in humans and mice, this paradigm of using non-coding RNA expression pattern to speculate putative novel gene function (simply based on expression alone) of well-studied genes is intriguing.

**Figure 1:**
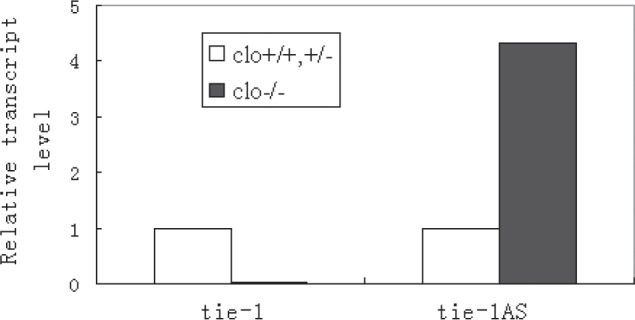
Real time PCR for tie-1 and tie-1AS across Clo^+/+^ and Clo^+/−^ or Clo^−/−^ embryos. Zebrafish *cloche* mutant embryos show aberrant level of *tie-1AS* compared with *tie-1*.

Mutations in Tie's have been previously implicated in patients with vascular malformations [31-35]. Our studies showed that the ratio of *tie-1* vs. *tie-1AS* lncRNA is altered in human vascular anomaly samples. The expression level of *tie-1AS* is much lower than that of *tie-1* in human placenta, but it goes up dramatically compared to the expression level of *tie-1* in hemangioma samples [36]. We observed similar expression discrepancy between *tie-1* and *tie-1AS* in zebrafish cardiovascular mutant *cloche* [37] (Figure 1). Interestingly, this data correlates well with previous reported [18] in situ hybridization profile for *tie-1* in *cloche* embryos where the expression is quite low when compared to WT embryos. The vascular anomaly (disease) and the *cloche* (genetic mutant) results together suggest that when the cognate gene level (*tie-1*) is low, the cognate *tie-1 AS* levels are high suggesting an inverse relationship between the *tie-1* and the AS transcript. In fact, this interpretation makes sense since we have shown the *tie-1AS* lncRNA selectively binds *tie-1* mRNA *in vivo* and regulates *tie-1* transcript levels [36]. Moreover, the normal expression levels of *tie-1AS* is a log lower than *tie-1* across different embryonic stages in zebrafish.

In most of the examples to date levels of antisense RNA have been correlated to disease. However, studies reporting genetic mutations in the antisense RNA are not common. A genetic mutation that gives rise to pathologic antisense transcript is a 14;18 chromosome translocation observed in most human follicular B-cell lymphomas. This translocation not only creates a bcl-2/IgH hybrid gene, but also gives rise to a bcl-2/IgH AS transcript. The AS transcript is merely present in the t(14;18)-positive, but not in the t(14;18)-negative lymphoid cell lines, and thus appears to be dependent on the bcl-2/IgH fusion [38]. Another recent example is observed in an individual with an inherited form of anemia (α-thalassemia). In this patient, the LUC7L transcription termination sequence is deleted and LUC7L RNA extends into HBA2 locus and causes CpG island methylation of HBA2, a α-globin gene in erythroid cells. The methylation leads to silencing of HBA2 gene resulting in an inherited form of anemia α-thalassemia [39] in this subject.

## NATS - PROMISING FUTURE APPLICATIONS

NATs hold immense potential for future applications. We will highlight three distinct areas of biology where NATs are expected to make promising contributions. More than 30 years ago, the inhibitory effects of antisense oligonucleotides on translation were observed *in vitro*. Since then synthetic antisense oligonucleotides have been used extensively to block target gene expression [40]. However, not all sites in mRNA sequences respond to targeting. Only one out of ten randomly selected antisense oligonucleotides exhibit good activity [41]. A study by Van der Krol et al using the petunia as a model to test the effects of antisense gene fragments corresponding to different chromosomal location on flower pigmentation highlights this point. The patterns of pigmentation varied among flowers of different transgenic petunia with sub-genomic fragments of antisense genes being ineffective in establishing a phenotype [42]. NATs are endogenous regulators targeting mRNA, and some of them are processed into endogenous siRNAs [43]. Thereby, NATs are ideal models for designing antisense oligonucleotides.

The second example highlights the role of NATs in a clinical trial setting. In clinical trials, an anticancer agent Gemcitabine can produce cytotoxic synergism with Cisplatin when given to cancer patients. It was proposed that the nucleotide excision repair (NER) process was responsible for the synergism. NER removes cisplatin-DNA adducts by a series of steps including damage recognition, dual incision/excision, repair synthesis and ligation. ERCC1 protein is an important factor in the incision process—the rate-limiting step of the pathway. Antisense therapy targeting the ERCC1 protein was proposed as a target for this approach. As a result, NER activity was downregulated and the cytotoxic synergism between Gemcitabine and cisplatin was abolished [44].

The third example highlights the role of NATs in tumor therapy. The bcl-2/IgH antisense transcript is presumed to be responsible for the overexpressed bcl-2, the root of oncogenicity in follicular lymphomas. Therapeutic ODNs targeting the bcl-2/IgH antisense transcript induced an early strong inhibition of cell growth and a late sudden cell death. The induction is restricted to follicular lymphomas which have t(14;18) chromosomal translocation, but not effective for the cells carrying a normal bcl-2 gene, suggesting tumor selectivity. However, antisense-oriented ODN, complementary to the bcl-2/IgH mRNA is ineffective [45].

## SUMMARY

NATs account for majority of non-coding RNAs in the vertebrate genome. Numerous mechanisms are utilized by NATs to regulate gene expression and these mechanisms span over both plant and animal kingdoms. Recently, NATs have been identified in various diseases as well and the regulation that they mediate when lost may in part be responsible for disease progression and pathogenesis. Although most studies identify dysregulation of NATs, very few studies have identified genetic mutations in them. As of now, NATs hold immense clinical value but their application is still at its infancy.
